# Provident Dispensaries

**Published:** 1899-10-21

**Authors:** 


### Provident Dispensaries.

Without making too much of the conference organised last week by the Hospital Reform Association it may well serve as an opportunity for considering the inter-relation of the several points discussed. Of the necessity of instituting some system of inquiry into the circumstances of the applicants for hospital treatment everyone seems to be agreed, but beyond this lies the question, what to do with the patients when inquired into. It must not be forgotten, although a tendency to forget it keeps cropping up in most unexpected quarters, that, after all, our hospitals are primarily and in their very essence charities, and that charity will always be ready to step in and provide what is not provided from other sources. Mr. Timothy Holmes said with great force that the most objectionable feature of the out-patient departments of hospitals was that they were entirely without efficient inquiry into the circumstances of the patients, and he went so far as to say that the hospitals took their charity and pitched it into the street for anyone to scramble after. This is a serious accusation; but it is idle to rail against such things until other means are provided by which medical help may be obtained by those just above the hospital class. Provident dispensaries are the means now suggested.

Unfortunately the provident dispensary system has failed to do what it was hoped at one time it might do, namely, fill the gap existing between the "real object of charity," who ought to go to hospital, and the "private patient," out of whose fast diminishing half-crown fee the general practitioner is imagined to live the life of a gentleman and to meet all the calls upon his purse incidental to such a position; and the cause of its failure lies to a large extent in the fact that its charitable nature has been overlooked. For, indeed, if kept to its proper work, the provident dispensary should really be a charity. But it is very difficult to keep a provident dispensary to its proper work. Its basis is payment, insufficient as that payment may be; and its contact with commercialism leads it into commercial ways. Money has to be collected, collectors have to be employed, and these collectors, being but human, spread their work among those who pay most easily—the very people from whom working class practices draw their income, people who never ought to be admitted to such dispensaries at all—and thus in some cases the whole affair becomes a mere business, whose very object would seem to be the provision of contract medical attendance at the lowest possible price.

In his paper on the Metropolitan Provident Medical Association, Mr. Warren said that from 30,000 to 40,000 persons were enrolled as provident members of its various branches, and that of their contributions £4,649 went to the medical men, including in this sum the expenses of drugs and dispensing. That is about 2s. 7d. a year for each provident member, or, deducting the dispensing and the drugs, perhaps about a halfpenny a week. Clearly this institution, so far as the payments to the doctors are concerned, is a charity, and its members require just as much "inquiry" as the out-patients of any hospital. So far as the question of provident dispensaries touches that of hospital reform, everything depends upon whether the patients who use these dispensaries are derived from the out-patient departments, or are recruited from among those who, but for the establishment of these dispensaries, would still be the private patients of general practitioners. Mr. Warren looks with satisfaction upon the £4,649, minus the expenses of drugs and dispensing, which is annually distributed among the "hundred or more" medical officers attached to the various branches of the Metropolitan Provident Medical Association, and says that "It is not unreasonable to assume that but for these provident dispensaries only a small proportion of this amount would find its way into the pockets of the general medical practitioners." But he gives no grounds for this assumption, and we confess we see none. Coincidently with the development of this large clientèle by the provident dispensaries, the hospitals have enormously increased the numbers of their out-patients, and glad as one would be to believe that the provident dispensary system afforded a way of escape from the difficulties of the hospitals, we see nothing to show that it has so far done anything of the sort. If managed on the basis of being a charity, and with all the restrictions that ought to be exercised in the distribution of charity, such dispensaries might indeed bridge over the gap in an admirable manner; but that has not yet been done—has not even been attempted. The fact has to be recognised that provident dispensaries are only intended for the poor. When, then, it is proposed to cure the evils connected with the out-patient system of our great hospitals by passing their patients on to the provident dispensaries, care must be taken that while abuse is remedied in one direction it is not created in another. The inquiry system which is now asked for in the hospitals is just as much wanted in the provident dispensaries.

